# Glandular Odontogenic Cyst: A Diagnostic and Management Dilemma

**DOI:** 10.7759/cureus.20701

**Published:** 2021-12-26

**Authors:** Senthilmurugan M, Senthilnathan Periasamy, Santhosh P Kumar, Ramvihari Thota

**Affiliations:** 1 Oral and Maxillofacial Surgery, Saveetha Dental College and Hospital, Chennai, IND

**Keywords:** glandular odontogenic cyst, jaw cyst, recurrence, resection, sialo-odontogenic cyst, mandible, odontogenic cyst

## Abstract

The glandular odontogenic cyst (GOC) is a rare odontogenic cyst that can develop in the maxillofacial region with aggressive behavior. It tends to develop into enormous proportions with high recurrence rates. The mandibular anterior area is the common site of occurrence of GOC, and it appears as an asymptomatic slow-growing swelling. GOC mimics other odontogenic cysts and tumors both clinically and radiographically, thus posing difficulty in diagnosis. Due to the aggressive potential of GOC, precise diagnosis and prompt treatment are crucial. Both conservative and aggressive surgical therapies have been advocated for GOC with a preference for aggressive therapy due to its high potential for recurrence. In this report, we present a case of GOC of the mandible in an adult female patient, which was successfully treated by segmental resection and primary reconstruction with stainless steel recon plate with uneventful healing during the one-year postoperative follow-up period.

## Introduction

The glandular odontogenic cyst (GOC) is a rare odontogenic cyst that can develop in the maxilla or mandible and is aggressive [1]. It tends to develop into enormous proportions with high recurrence rates [2]. Padayachee and Van Wyk originally described it as a "sialo-odontogenic cyst" in 1987 [3]. Gardner et al. [4] characterized it as a unique clinicopathologic entity and coined the term "glandular odontogenic cyst" due to the odontogenic nature of the epithelial lining of the cyst. World Health Organization (WHO) classified it as a sialo-odontogenic cyst or glandular odontogenic cyst in the WHO histological classification of odontogenic tumours [5].

The mandibular anterior area is the common site of occurrence of GOC, which presents as a painless, slow-growing mass. GOC typically affects people in the fourth and fifth decades, with male predisposition [6]. The radiographic appearance of GOC is non-specific, and it may present on radiographs as a unilocular or multilocular radiolucency with well-defined and scalloped borders [7]. As GOC exhibits multilocularity in the mandibular posterior region, differential diagnosis of GOC includes odontogenic keratocyst, aneurysmal bone cyst, low-grade mucoepidermoid carcinoma, ameloblastoma, central giant cell granuloma, and radicular cyst [8]. There is no discernible difference in radiological findings between the glandular odontogenic cysts and other odontogenic cysts, central mucoepidermoid carcinoma. Only histopathological examination can differentiate between glandular odontogenic cysts and other odontogenic cysts, central mucoepidermoid carcinoma [9,10].

Curettage and enucleation are common treatments for GOC. Because glandular odontogenic cysts are clinically aggressive with a potential for recurrence, resection of the jaw with longer follow-up is preferred [11]. In this report, we present a case of GOC of the mandible in an adult female patient, which was successfully treated by segmental resection and primary reconstruction with stainless steel recon plate.

## Case presentation

**Figure 1:**
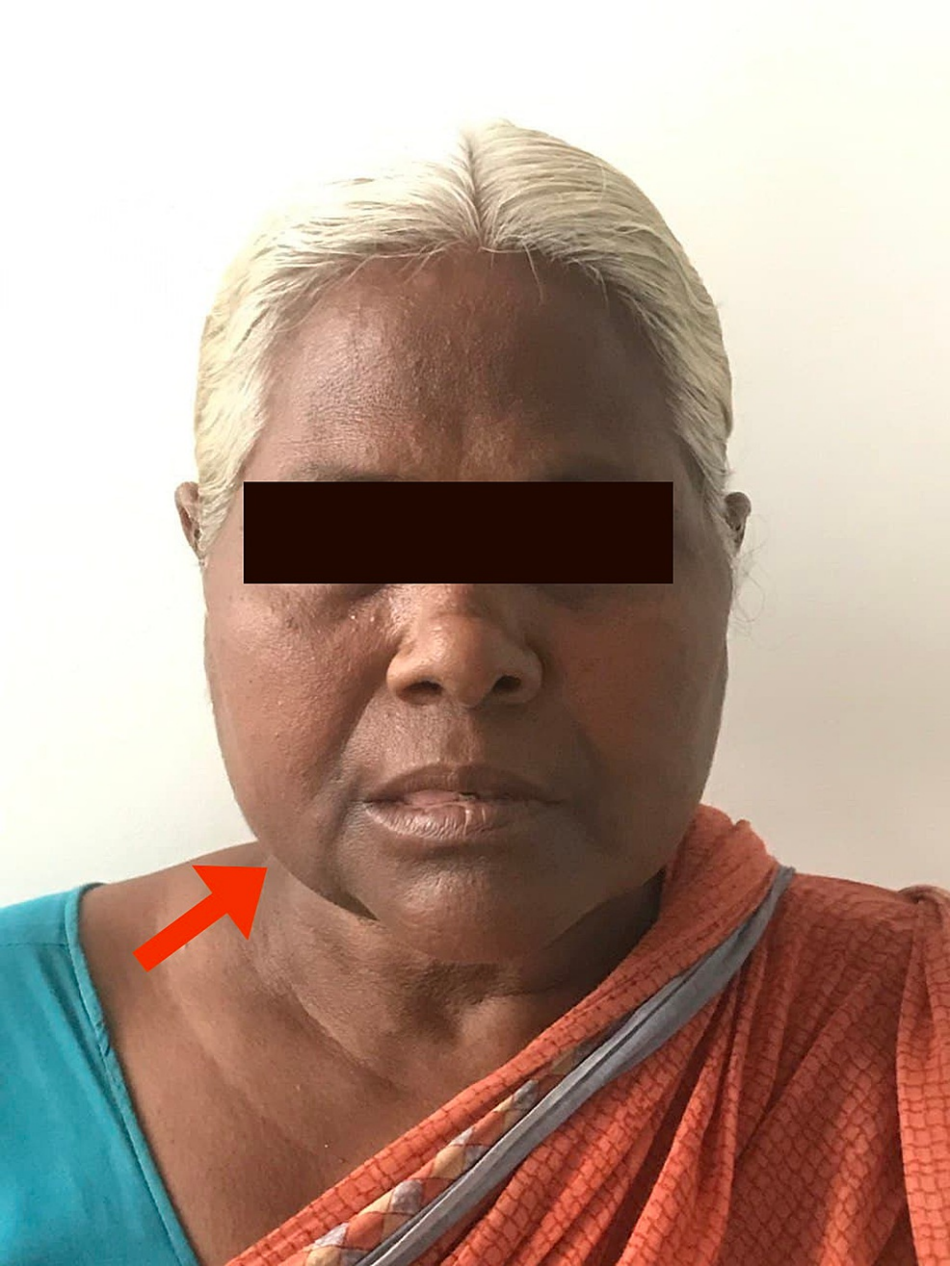
Extraoral view showing swelling on the right side of the face.

**Figure 2:**
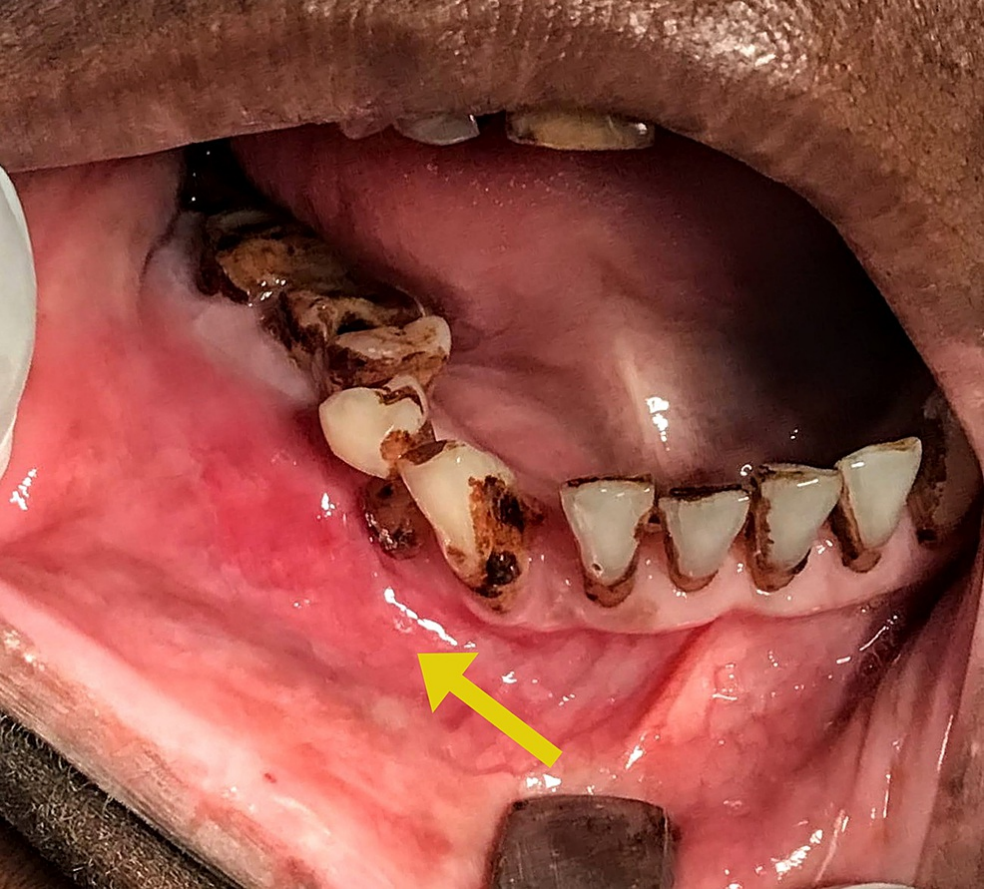
Intraoral view shows obliteration of the vestibule due to the presence of the lesion.

A 60-year-old female patient presented to the department of oral and maxillofacial surgery with a chief complaint of painless swelling over the right side of the face for four years (Figure 1). History revealed that the swelling gradually increased to the present size, and the patient did not have any functional problems. On the right cheek, the swelling was approximately below the mid-cheek region; measuring about 5 cm×4 cm, and skin over the lesion was normal. The swelling was extending superoinferiorly from the level of the commissure of the lip to the lower border of the mandible and anteroposteriorly 1.5 cm from the right angle of mouth to 1 cm short of the right angle of the mandible. Palpation revealed a firm, non-tender, non-pulsatile swelling which was fixated to the underlying structures without well-defined margins. Intraorally, there was the obliteration of the vestibule from 43 to 48 (Figure 2). No pus discharge or any inflammatory fluid was present. 

**Figure 3:**
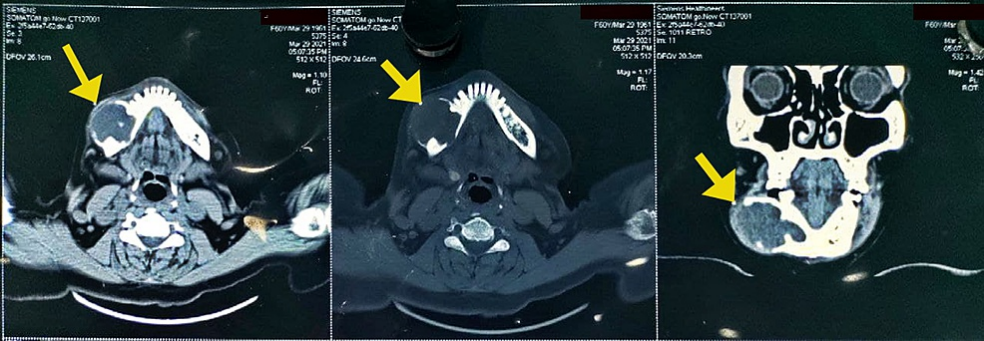
CT scan sections showing well-defined encapsulated homogeneously enhancing lesions in the right body of the mandible.

The patient’s blood parameters were normal. A computerized tomography (CT) scan revealed a well-defined lesion in the right body of the mandible from 43 to 48 with buccolingual cortical expansion and perforation sparing the adjacent structures (Figure 3). Ameloblastoma, odontogenic keratocyst, and aneurysmal bone cyst were included in the differential diagnosis of the lesion.

**Figure 4:**
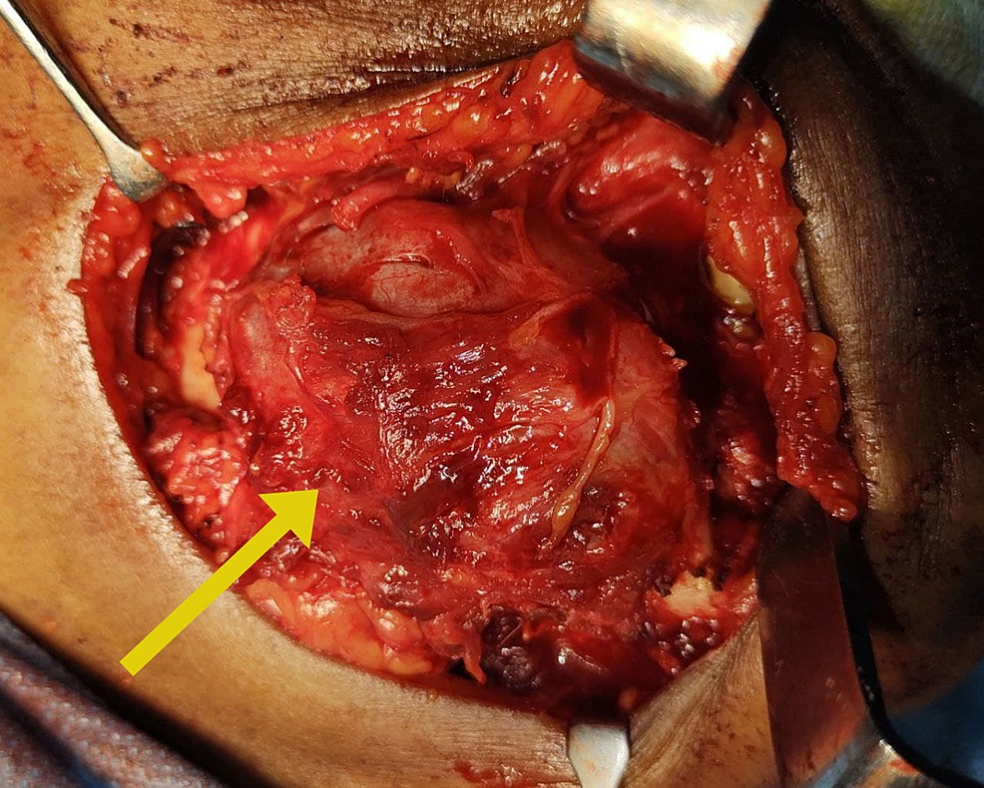
Glandular odontogenic cyst in the mandible

**Figure 5:**
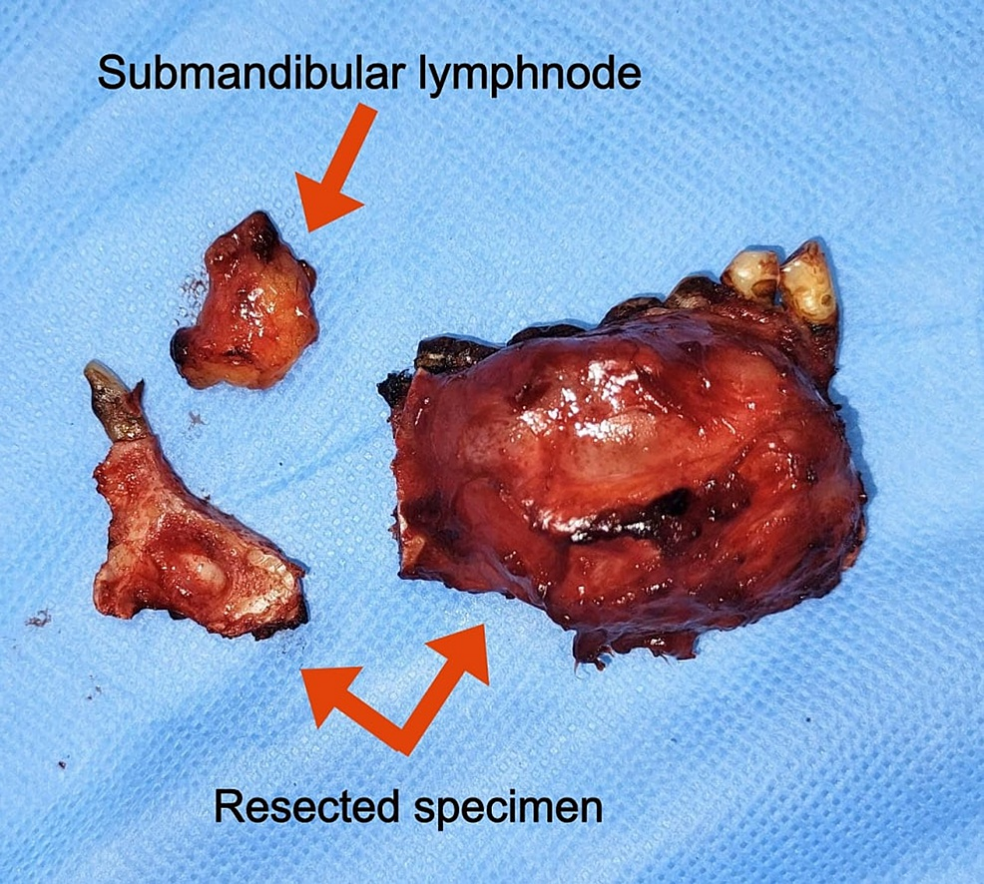
Glandular odontogenic cyst removed in toto along with the lymph nodes

**Figure 6:**
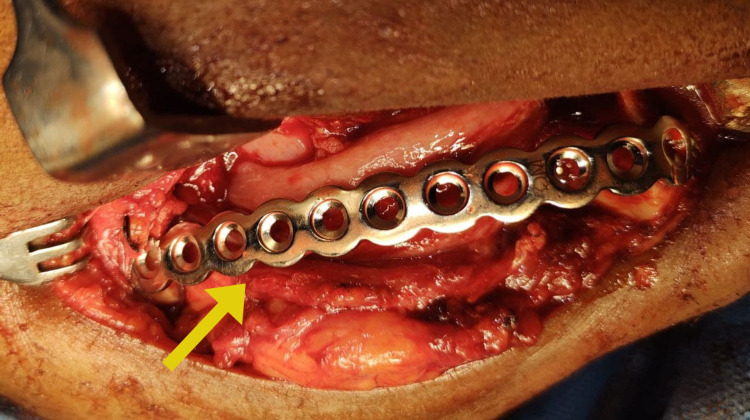
Primary reconstruction of the surgical defect with fixation of stainless steel recon plate.

Fine needle aspiration cytology was performed, which revealed a thick cheesy, greyish white fluid, and the samples were sent for histological examination with provisional diagnosis as odontogenic keratocyst. Incisional biopsy was performed, and the histological examination revealed features suggestive of a glandular odontogenic cyst. With a confirmatory diagnosis of the glandular odontogenic cyst, the decision was made to surgically resect the cystic lesion in the mandible with safe margins under general anesthesia. With an extended submandibular approach, blunt dissection was done, the vital structures were secured and, the mandible along with the lesion was exposed (Figure 4). Segmental resection of the mandibular segment from the 42 regions to the right angle of the mandible with safe margins of 1 cm both anteriorly and posteriorly was done along with excision of the submandibular lymph nodes (Figure 5). The surgical defect was primarily reconstructed using stainless steel recon plate fixation (Figure 6).

**Figure 7:**
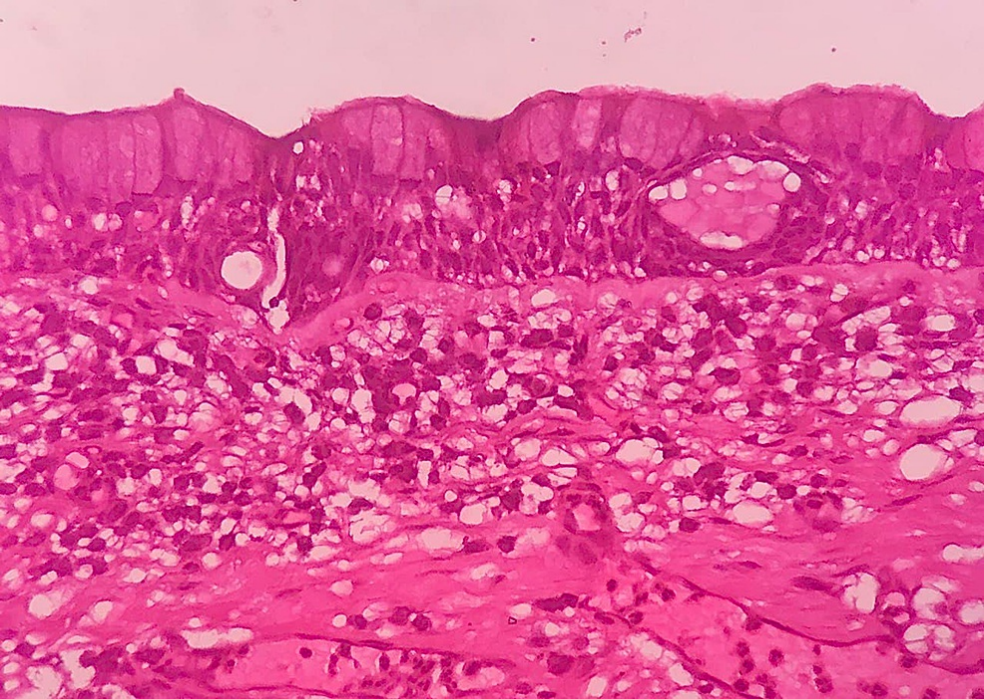
Histopathological image showing odontogenic epithelial lining and connective tissue wall with features typical of the glandular odontogenic cyst.

The resected specimen consisting of lesions of size 5.1x2.9x3 cm was sent for histopathological examination. Histopathological examination showed predominantly non-keratinized stratified epithelium, microcysts, and duct-like structures in the odontogenic epithelial lining. Moderate vascularity, skeletal muscle, nerve bundle, adipose tissue, and peripheral resorting bone were also present, confirming the diagnosis of a glandular odontogenic cyst (Figure 7).

**Figure 8:**
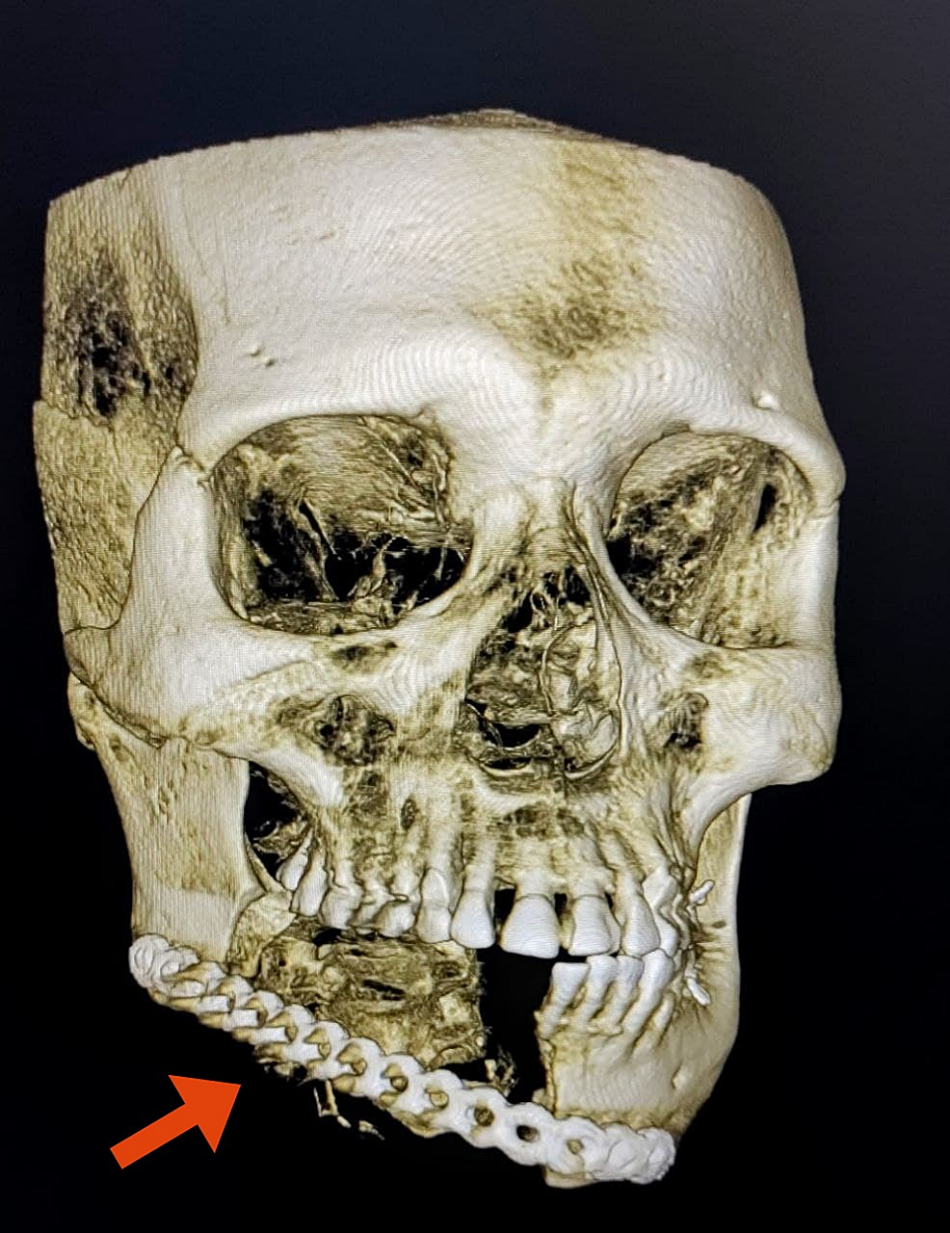
Cone-beam computerized tomography scan revealing stability of the recon plate over the defect at one-year postoperative follow-up period.

Clinical examination during a one-year postoperative follow-up period showed facial symmetry and good wound healing with no signs of recurrence. Cone-beam computerized tomography (CBCT) revealed good adaptation of the recon plate over the defect (Figure 8). 

## Discussion

The genesis of GOC remains unknown, although numerous cases of hybrid lesions of GOC occurring with other odontogenic cancers have been reported. Immunohistochemistry analysis, on the other hand, does not indicate a sialogenic origin [12]. GOC is a rare variant of the odontogenic cyst with atypical histopathological presentation. Histologically GOC exhibits features similar to a botryoid odontogenic cyst, radicular cyst with mucus prosoplasia, lateral periodontal cyst, dentigerous cyst, as well as mucoepidermoid cancer. GOC must be considered in the differential diagnosis of radiolucent lesions of the jaws [11]. Due to the aggressive potential of GOC, precise diagnosis and prompt treatment are crucial for a favorable prognosis [13].

Most of the GOCs reported in the literature are asymptomatic, with the majority in the mandible (70%) rather than maxilla (30%) [13]. GOC in our patient presented as a painless lesion in the right side of the mandible. GOC manifests in varying sizes (mean diameter, 4.9 cm) and important characteristics to be considered in the differential diagnosis of GOC are the locularity, radiodensity, and border features [14]. GOC is exhibited as a well-defined unilocular or multilocular radiolucent lesion on radiograph linked to tooth impaction or displacement, as well as root resorption. Most GOCs exhibit expansion with thinning, erosion, or perforation of the cortical plates. There is no pathognomonic radiologic characteristic for GOCs. Unicystic or multicystic ameloblastoma, lateral epithelial cyst (LPC), odontogenic keratocyst, and central mucoepidermoid tumor all have the same radiologic characteristics as glandular odontogenic cyst [7]. GOC closely resembles ameloblastoma, and features like uneven loculation, sclerotic boundary, and perforation can help to differentiate GOC from ameloblastoma [8]. Our patient exhibited a unilocular radiolucency of the mandible, thinning, and erosion of the cortical plates with the expansion of the mandible.

According to the literature, some microscopic criteria should be rigorously used to avoid an erroneous diagnosis (such as odontogenic keratocyst or ameloblastoma) [4]. Features typical of GOC include the presence of glandular or pseudo glandular structures, cuboidal or columnar epithelial cells, and mucin containing intraepithelial crypts. Multicystic type displays neoplastic characteristics (infiltration of surrounding tissue and/or daughter cyst development) more than the unicystic type. More research is needed to declare that GOC and mucoepidermoid carcinoma of the jaws are two different diseases. Although both exhibit similar clinical and radiographic features, the presence of epithelial plaques is unique to GOC [7].

Several therapy approaches for GOC have been proposed in the literature, both conservative and aggressive [15]. Conservative treatment modalities considered for GOC include marsupialization, enucleation, curettage with adjuvant Carnoy's solution or cryotherapy, curettage with and without peripheral ostectomy. Due to the high recurrence rate of GOC, aggressive treatment modalities ranging from marginal resection to segmental jaw resection are recommended in the management of GOC and have yielded successful outcomes [12-15]. Big multilocular GOCs are aggressive enough to warrant aggressive surgical therapy. Enhanced Ki-67 positivity and decreased p53 positivity are linked to the strong proliferative behavior of GOC [16]. The presence of microcysts and the thinness of the cyst are attributed to the high recurrence rate of GOC. It was reported that the recurrence rate was nil with aggressive resection in comparison to conservative approaches which showed recurrences [13].

The size and locularity of this cystic lesion are directly connected to the recurrence rate, regardless of the therapeutic technique utilized. The high recurrence rate of GOCs might be linked to partial cyst clearance due to the cyst's multicystic structure, the epithelium's inclination to detach from the connective tissue, or the cyst's development via the cancellous areas of bone [15]. It was reported that most patients with recurrent illness had extensive multilocular lesions with cortical perforations. After excision, they advocated a rapid repair using a free fibula flap to restore the mandibular bone height and allow oral rehabilitation with endosseous implants. Resection of the overlying mucosa is also recommended in GOCs with cortical perforation [17,18]. Thus, choosing an aggressive surgical strategy to avoid recurrences can lead to successful outcomes. 

## Conclusions

GOC is a rare variant that mimics other odontogenic cysts and tumors both clinically and radiographically, thus posing difficulty in diagnosis. Due to the aggressive potential of GOC, precise diagnosis and prompt treatment are crucial. The management of GOC is also challenging due to its aggressive nature and the tendency to recur. Although both conservative and aggressive surgical therapies have been advocated for GOC, aggressive surgical resection with immediate reconstruction is implicated for successful outcomes. Nonetheless, it is critical to evaluate the patients' overall clinical circumstances as well as their therapeutic preferences. Every patient with GOC should be closely monitored for early detection of recurrences.
